# Diagnostic Value of Unenhanced CT Attenuation and CT Histogram Analysis in Differential Diagnosis of Adrenal Tumors

**DOI:** 10.3390/medicina56110597

**Published:** 2020-11-09

**Authors:** Paulína Szász, Petr Kučera, Filip Čtvrtlík, Kateřina Langová, Igor Hartmann, Zbyněk Tüdös

**Affiliations:** 1 Department of Radiology, University Hospital and Faculty of Medicine and Dentistry, Palacky University, 779 00 Olomouc, Czech Republic; paulina.szasz@fnol.cz (P.S.); petr.kucera@fnol.cz (P.K.); filip.ctvrtlik@fnol.cz (F.Č.); 2 Department of Medical Biophysics, Faculty of Medicine and Dentistry, Palacky University, 775 15 Olomouc, Czech Republic; katerina.langova@upol.cz; 3 Department of Urology, University Hospital and Faculty of Medicine and Dentistry, Palacky University, 779 00 Olomouc, Czech Republic; igor.hartmann@fnol.cz

**Keywords:** adrenal gland neoplasms, adrenocortical adenoma, computed tomography, histogram analysis, adrenal incidentaloma

## Abstract

*Background and Objectives*: Our aim was to verify the optimal cut-off value for unenhanced CT attenuation and the percentage of negative voxels in the volume CT histogram analysis of adrenal masses. *Materials and Methods*: We retrospectively analyzed the CT data of patients who underwent an adrenalectomy in the period 2002–2019. In total, 413 adrenalectomies were performed. Out of these, 233 histologically verified masses (123 adenomas, 58 pheochromocytomas, 18 carcinomas, and 34 metastases) fulfilled the inclusion criteria and were selected for analysis. The mean unenhanced attenuation in Hounsfield units (HU) and the percentage of voxels with attenuation less than 0 HU (negative voxels) were measured in each mass. *Results*: The mean unenhanced attenuation with a cut-off value of 10 HU reached a sensitivity of 59.4% and a specificity of 99.1% for benign adenomas. The mean unenhanced attenuation with a cut-off value of 15 HU reached a sensitivity of 69.1% and a specificity of 98.2%. For the histogram analysis, a cut-off value of 10% of negative pixels reached a sensitivity of 82.9% and a specificity of 98.2%, whereas a cut-off value of 5% of negative pixels reached a sensitivity of 87.8% and a specificity of 75.5%. The percentage of negative voxels reached a slightly better area under the curve (0.919) than unenhanced attenuation (0.908). *Conclusion*: Mean unenhanced attenuation with a cut-off value of 10 HU represents a simple tool, and the most specific one, to distinguish adrenal adenomas from non-adenomas. CT histogram analysis with cut-off values of 10% of negative voxels improves sensitivity without any loss of specificity.

## 1. Introduction

With the increasing availability of imaging methods, the probability of detection of an adrenal mass is also increasing. Their prevalence is reported as being up to 10% [1]. In clinical practice, it is important to decide whether the mass is benign or appears to be indeterminate or malignant and whether it is necessary to take an active approach to the management of an adrenal mass.

The basic assessment is the measurement of its unenhanced attenuation in Hounsfield units (HU). Masses with an unenhanced attenuation of less than 10 HU could be considered benign [2,3,4]. Unfortunately, about a third of adenomas have an unenhanced attenuation higher than 10 HU [4] and should be considered indeterminate. A CT histogram analysis was proposed to distinguish these adenomas, which are termed lipid-poor, from non-adenomas [5]. This analysis is based on the calculation of the portion of the pixels inside the adrenal mass with an attenuation of less than 0 HU, i.e., the percentage of negative pixels.

The method has already been verified in several papers, but most of these articles dealt with a relatively low number of patients and only a small portion of the tumors was verified histologically [6,7,8,9]. In addition, all the published papers used a single-image analysis, although the assumption is that a volume histogram should achieve better accuracy [10].

The aim of our study was to verify the diagnostic value of unenhanced attenuation and volume histogram analysis on a large set of histologically verified adrenal tumors.

## 2. Materials and Methods

### 2.1. Study Design

The available CT data of patients who underwent an adrenalectomy in a single tertiary center during the time period 2004–2019 were analyzed retrospectively. 

The inclusion criteria were (1) an adrenal mass with a size > 10 mm, (2) an available unenhanced CT scan, and (3) a final diagnosis based on histology. The ethics committee approved the study; informed consent was waived.

### 2.2. Study Group

In total, 413 adrenalectomies were performed in our institution during the time period 2004–2019. In 103 of them, an unenhanced CT scan was unavailable (e.g., MRI, PET/CT, only contrast-enhanced CT scans or printed sheets were available in these subjects). Macroscopically normal adrenal glands or lesions smaller than 10 mm were found in 45 subjects. Further, we excluded eight cystic lesions, seven pseudocystic lesions, three rare tumors (one liposarcoma, one PEComa, and one schwanoma), and two adrenal glands directly infiltrated by renal cell carcinomas. Finally, we decided to exclude 12 myelolipomas since they were benign and did not need to be strictly distinguished from non-functional adenomas. Therefore, retrospective analysis was performed on 233 masses in 226 subjects. The study group included 101 males and 125 females; the age range was 18–83 years and the mean age was 55.6 years. The adrenal masses were located on the right side in 104 cases, on the left side in 114 cases, and bilaterally in eight cases. The study group included 123 adenomas; the remaining adrenal masses were non-adenomas (58 pheochromocytomas, 34 metastases, and 18 adrenocortical carcinomas).

### 2.3. CT Protocols

In our institution, CT examinations were performed on several CT scanners (HiSpeed Advantage CT/i or LightSpeed RT16 or LightSpeed VCT or Discovery CT750HD, GE Healthcare, Milwaukee, WI, USA). Various protocols were applied using the usual routine settings for the imaging of the thoracic and abdominal organs. A minority of subjects were referred from other hospitals. In these patients, CT data were obtained with scanners from multiple vendors using different scan protocols. The exposure parameters collected in all subjects from archived “digital imaging and communications in medicine” records were as follows: The tube voltage mean was 120.86, the median 120, and the range 110–140 kV; the tube current mean was 364.38, the median 298, and the range 140–799 mAs; and the slice thickness mean was 4.76, the median 5, and the range 1.5–7 mm. The field of view (FOV) was adjusted individually to cover the patient’s body and the matrix was 512 × 512. A “soft” reconstruction kernel was used in all our studies.

### 2.4. CT Image Analysis

**Figure 1:**
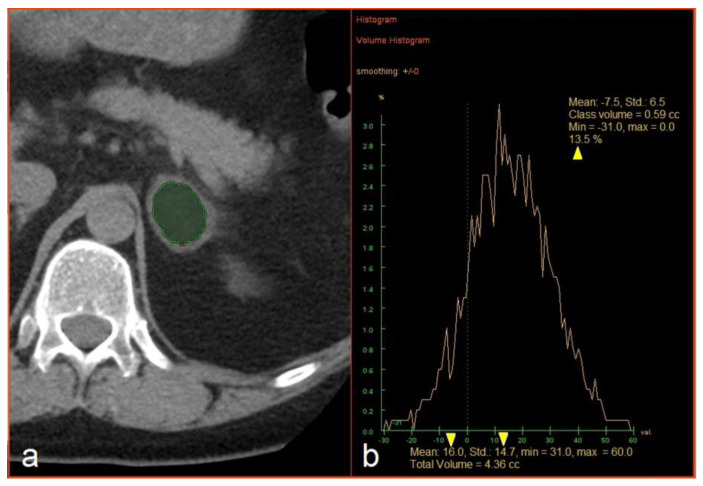
(**a**) Manual extraction of an adrenal mass. (**b**) An example of histogram analysis and extraction of the mean attenuation, the standard deviation of Hounsfield values, and the percentage of negative voxels (yellow arrowheads).

The CT images were loaded into an Advantage Windows workstation v4.4 supplied by a CT vendor (GE Healthcare, Milwaukee, WI, USA). The evaluation included the measurement of three dimensions of each lesion and hand-traced slice-by-slice extraction of the volume of interest (VOI) inside a lesion covering as large a part of the soft tissue volume as possible while avoiding the edges and any necrotic parts of a lesion. The mean attenuation and standard deviation (SD) of the HU values were recorded, as was the percentage (%) of voxels with attenuation <0 HU, in a histogram using the “Volume Histogram” tool (Figure 1). The analysis was performed by PK (2 years of experience) and PS (6 years of experience) and supervised by FČ (16 years of experience); the results of the two researchers were combined to create one dataset. All three radiologists were blinded with respect to the final diagnosis.

### 2.5. Statistical Analysis

Shapiro–Wilk tests of normality revealed non-normal distribution of the data in all selected variables. The data were expressed as median, minimal value, maximal value, mean, and standard deviation. Further, a post-hoc Mann-Whitney U-test with Bonferroni correction was used to compare four independent groups (adenomas, pheochromocytomas, carcinomas, and metastases). The value of *p* < 0.05 was adopted as the level of statistical significance. Receiver operating characteristic (ROC) was performed to obtain ROC curves and the value of the area under the curve (AUC). Finally, we calculated the sensitivity (Sens), specificity (Spec), and positive predictive value (PPV) for the diagnosis of an adenoma.

All statistical analyses were conducted with IBM SPSS Statistics for Windows, Version 23.0. Armonk, NY, USA: IBM Corp.

## 3. Results

**Figure 2:**
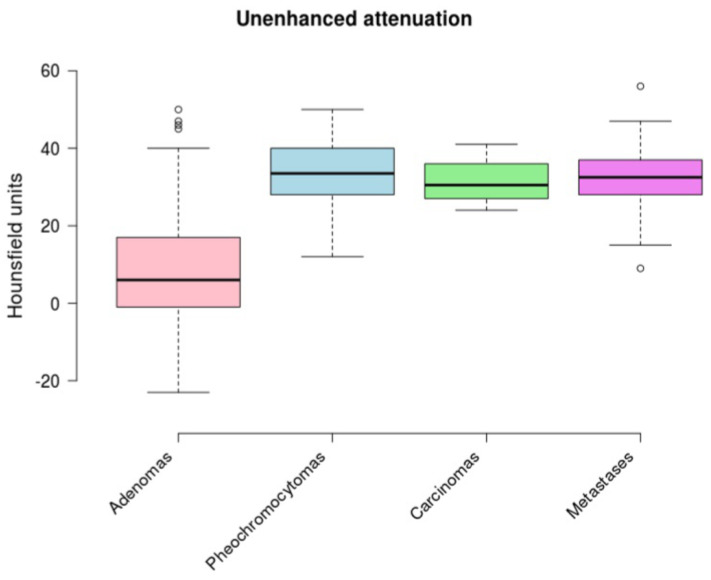
A boxplot comparing unenhanced attenuation between the groups.

**Figure 3:**
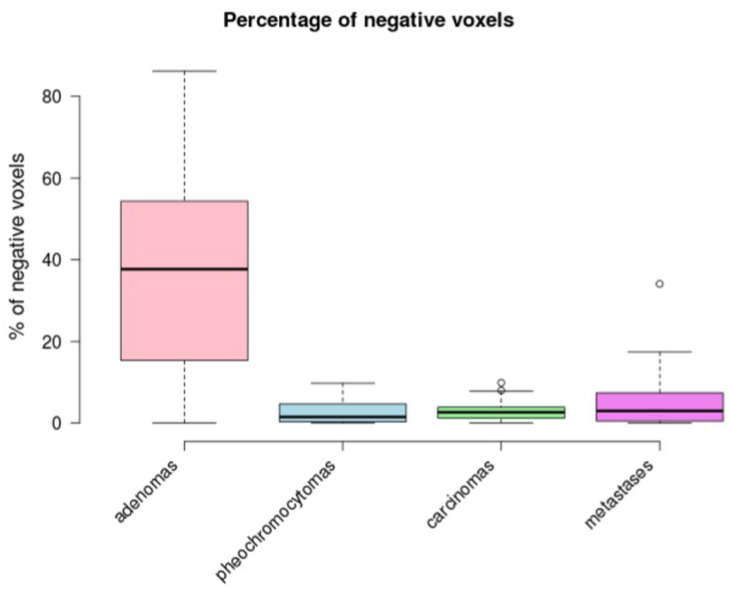
A boxplot comparing the percentage of negative voxels between the groups.

Our data demonstrated a statistically significant difference in the mean unenhanced attenuation between adenomas and non-adenomas ((a) in Table 1, and Table 2). There was no statistically significant difference between pheochromocytomas, carcinomas, and metastases (Table 2). We obtained the same result for the percentage of negative voxels ((b) in Table 1, and Table 2). The results are shown graphically in Figure 2 and Figure 3. 

On the other hand, the differences between the groups in terms of the SD of HU values as a measure of CT image noise were insignificant (Table 1). Therefore, the differences in the percentage of negative voxels could not be explained by different levels of image noise.

**Figure 4:**
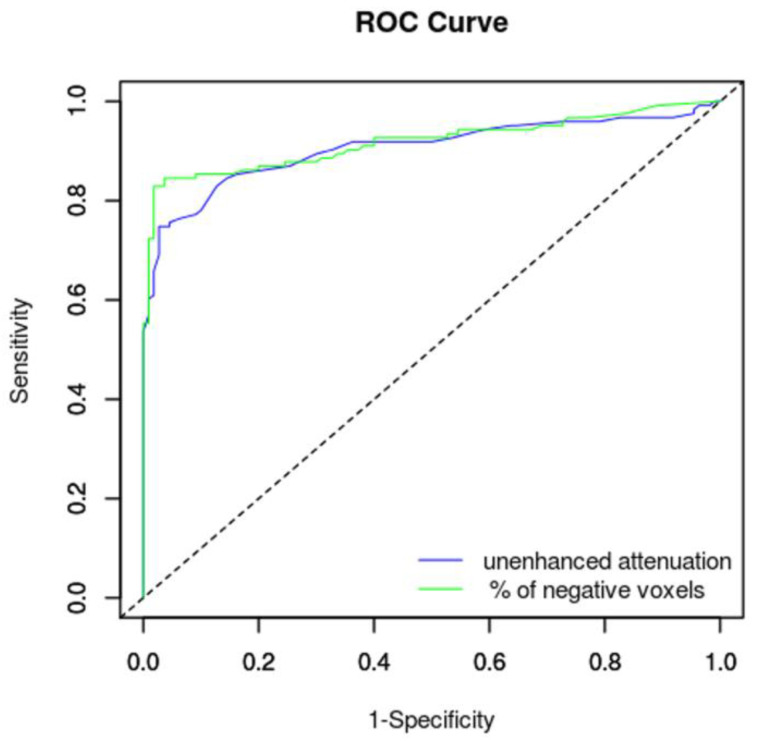
Receiver operating characteristic curves of an unenhanced attenuation and percentage of negative voxels with respect to diagnosis of an adrenal adenoma.

The AUC of unenhanced attenuation and percentage of negative voxels reached excellent diagnostic values; the percentage of negative voxels reached a slightly better result (Table 3). The ROC curves are shown in Figure 4.

Sensitivity, specificity, and PPV values for unenhanced attenuation using 10-HU and 15-HU cut-off values and the percentage of negative voxels using 5% and 10% cut-off values are shown in Table 4.

## 4. Discussion

Adrenal focal masses represent an important challenge in clinical practice. Whether one is discovered incidentally, during a staging, or at the request of an endocrinologist, all the differential diagnostic alternatives should always be carefully considered [1,11,12].

For the subsequent clinical management, it is important to decide whether a lesion is benign, indeterminate, or displays clear signs of malignancy, and whether it is necessary to take an active approach to the lesion. The aim of a radiologist is to reliably recognize as many benign lesions as possible without the need for extensive investigation to save the patient from ionizing radiation and the use of contrast media and, last but not least, the stress of uncertainty and follow-up examinations. In CT examination, the diagnosis of an adenoma is based on low unenhanced attenuation because of intracytoplasmic fat. An unenhanced attenuation of 10 HU is generally considered to be the crucial cut-off value between adenomas and non-adenomas [2,3,4]. In clinical practice, the simplicity of this measure is a great advantage, as is the fact that the measurement can be performed even when CT is not primarily aimed at the adrenal glands. Approximately 30% of adenomas, however, have an unenhanced attenuation higher than 10 HU [2,3,4]. Such adenomas are called “lipid-poor” and represent a significant clinical problem, because it is impossible to reliably distinguish lipid-poor adenomas from other adrenal masses by means of unenhanced CT. A CT histogram analysis is another possible way to analyze an initial unenhanced CT image.

The purpose of our study was to retrospectively evaluate a large sample of adrenal masses and to verify and compare the diagnostic accuracy of unenhanced attenuation and CT histogram analysis. Our group represents the largest group of adrenal masses evaluated by histogram analysis to be published so far. A great advantage of our work compared to previous studies is the histological verification of all the masses, as, with the exception of Remer et al. [13], the authors of previous articles reporting the results of CT histogram analysis used different reference standards for their final diagnosis, e.g., CT wash-out rates, chemical shift MRI, or PET/CT tracer accumulation. Unfortunately, these methods may be misleading, since the wash-out rate calculations may be false positive in pheochromocytomas or metastases [14,15,16,17]. Similarly, the specificity of MRI and PET/CT may not be sufficient [12], and histology is thus the only fully reliable standard of reference. Furthermore, the CT histogram calculation methodology was different compared to other previous works. We calculated a histogram in the volume of interest using multiple CT images, while previous papers reported the calculation of a CT histogram in the region of interest using a single CT image. Our assumption was that the volume histogram would be more representative and less affected by local mass heterogeneity [10]. 

One of the aims of our work was to verify that the 10-HU threshold is optimal or whether another threshold (e.g., 15 HU) could be more appropriate. Using a cut-off value of 10 HU, we achieved a sensitivity of 59.35% and a specificity of 99.09%; using a cut-off value of 15 HU, we achieved a sensitivity of 69.11% and a specificity of 98.18%. In their large meta-analysis, Boland et al. [4] reported a sensitivity of 71% and a specificity of 98% using a 10-HU threshold and a sensitivity of 80% and a specificity of 94.5% for a 14-HU threshold. The lower sensitivity in our group of surgically removed masses is probably due to the lower proportion of lipid-rich adenomas that need not be operated on if they are hormonally inactive. Our sample achieved better specificity using a 10-HU threshold because only one false positive result was found, in a metastasis of a renal cell carcinoma, in which we measured an attenuation of 9 HU. Another metastasis of a renal cell carcinoma reached a attenuation of 15 HU. Other non-adenomas had a mean unenhanced attenuation higher than 15 HU. Thus, both 10 HU and 15 HU have sufficient specificity.

A CT histogram analysis method was proposed by Bae et al. [5] to help identify lipid-poor adrenal adenomas. The principle of the analysis is to quantify the number of pixels with a density of less than 0 HU in a specified ROI on a CT scan. An unenhanced CT scan is most suitable for the analysis and it can be performed retrospectively, which is important in masses that are discovered incidentally. Our results summarized in Table 4 show that the threshold of 5% of negative voxels is slightly more sensitive compared to the 10% threshold (87.8% vs. 82.93%), but its specificity is significantly lower (75.45% vs. 98.18%), as is its PPV (98.08% vs. 80%). Therefore, a threshold of 10% of negative voxels is, in our opinion, more suitable for routine clinical practice because of its better specificity and PPV. In our group, only two non-adenomas exceeded 10% of negative voxels; interestingly, both of them were metastases of renal cell carcinomas.

In their original paper, Bae et al. [5] achieved a sensitivity of 94% using a cutoff of 5% of negative pixels and 92% with a cutoff of 10% of negative pixels; the specificity was 100% in both cases. It is noteworthy that 74 out of 90 adenomas (82.2%) in their group were lipid-rich. Most of the 31 metastases were lung cancer metastases and only one metastasis was histologically verified. A study published by Remer et al. [13] used a very similar methodology to ours. Their group contains the largest sample of histologically verified masses published so far (105 adenomas and 76 non-adenomas) using unenhanced attenuation. Their readers reached a sensitivity of 78.1–81% and a specificity of 67.1–76.3% at a cut-off value of 5% of negative pixels; using a cutoff value of 10% of negative pixels, the sensitivity was 69.5–72.4% and the specificity 85.5–89.5%. In this respect, our results have achieved better values. This could result from differences in the CT methodology, as Remer et al. used a slice thickness of 2.5 mm and 5 mm without reporting which one was more frequently used; if a 2.5-mm slice thickness was more frequent, the noise in such CT images would be higher. Additionally, the reported tube current was between 200 and 250 mAs, which is lower than in our study (median 298 mAs). The difference in exposure parameters could lead to a higher value of noise in the CT image and an artificial increase in negative pixels. In a study group consisting of 62 lipid-rich adenomas, 31 lipid-poor adenomas, and 36 lung cancer metastases, Ho et al. [6] achieved a 100% sensitivity for lipid-rich adenomas and a 51% sensitivity for lipid-poor adenomas using a threshold of 10% of negative pixels; the specificity reached 100%. However, only a minor proportion of tumors were histologically verified. Halefoglu et al. [8] achieved a sensitivity of 91% and a specificity of 100% using a threshold of 10% of negative pixels; however, their results indicate that the vast majority of their adenomas were lipid-poor and only a few of the adenomas had an unenhanced attenuation more than 15 HU. Their non-adenoma group contained only metastases. Another recent paper regarding CT histogram analysis is a paper by Lin et al. [9] containing 131 benign and 43 malignant masses. What is problematic in this article is the methodology for identifying benign and malignant masses, as it used, for example, CT wash-out rates, MRI, or PET/CT tracer accumulation as a reference standard, and only nine masses were histologically verified. Comparing the results with other works, including ours, is not straightforward, since their study groups included only indeterminate lipid-poor masses, so their sensitivity is less than 48% at a cutoff value of 10% of negative pixels. However, they achieved a high specificity value of 98%, which was certainly increased by the elimination of high-noise CT scans. Similar results were reported by Jhaveri et al. [7] in their sample of 28 lipid-poor adenomas and 11 non-adenomas (mostly lung cancer metastases) using a threshold of 10% of negative pixels; a sensitivity of 46% and a specificity of 100% were reported. Very similar results have been achieved in our previous work in lipid-poor indeterminate masses [18]. The most recent paper published on the topic of histogram analysis is by Clark et al. [19]. In their group of 33 adenomas and 33 non-adenomas, CT histogram analysis using a cut off value of 10% of negative pixels reached a sensitivity of 84.8% and a specificity of 42.4%. There is again a significant difference in exposure parameters compared to our study; Clark et al. reported a median slice thickness of 2.5 mm, a median tube current of 199 mAs, and a range between 40 and 643 mAs. These parameters led to higher noise in the CT images, which the authors revealed in their article—the average SD of CT values was 24.8 ± 10.4 HU (25.7% had a standard deviation ≥ 30 HU). It should also be noted that the authors’ goal was to prove that their noise-corrected Gaussian model-based algorithm would achieve a better result than CT histogram analysis based on counting negative pixels, so it is possible that the authors appreciated the higher image noise, which could then be eliminated by their noise-corrected formula [19].

As already mentioned, the noise in the CT image has a significant effect on the number of negative voxels [20]. The simplest and most readily available parameter that allows the estimation of the CT image noise is the SD of CT values inside the region of interest. Lin et al. were the first to publish this methodology in connection with histogram analysis [9]. According to their measurements, CT images with an SD higher than 30 HU are too noisy and inappropriate for CT histogram analysis. In our previous article, we confirmed that the specificity of the CT histogram analysis decreases significantly and there is a significant increase in false positive results in non-adenomas if the SD is more than 30 HU [18]. We calculated and recorded the SD in each lesion; the median and mean of the SD of CT values in our recent study group ranged from 14.6 to 17.5 HU, which is safely below the threshold of 30 HU. Therefore, we can state that the noise was within a range that is unlikely to lead to false positive results, which is reflected by the very high specificity, which is comparable to that described in previous papers [5,6,8].

Another approach to eliminating the influence of noise from CT histogram analysis was published recently; Clark et al. proposed the use of a Gaussian model-based algorithm with noise correction to characterize adrenal tumors [19]. Their method uses a formula that includes the average density, SD, tube current, tube voltage, slice thickness, and pitch, which allows the estimation and mathematical elimination of noise [19]. This approach seems suitable in, e.g., low-dose CT examinations performed with a low tube current and high CT image noise.

On the basis of our results and previous papers, it can be stated that all lipid-poor adenomas contain more than 10% of negative voxels, while only about half of lipid-poor adenomas exceed this threshold [6,7,8,9,18]; thus, the sensitivity of histogram analysis mostly depends on the proportion of lipid-poor and lipid-rich adenomas in the study group. The specificity of a threshold of 10% of negative voxels is virtually 100%, provided that the noise in the CT image is taken into account [9,18]. Non-adenomas with unenhanced attenuation less than 10 HU or with more than 10% of negative voxels are very rare. There were two such tumors in our group, both of which were metastases of renal cell carcinomas. The most common lung cancer metastases usually do not exceed a limit of 10% of negative voxels [5,6,7]. Using a threshold of 5% of negative voxels increased the sensitivity by about 5%, but the specificity decreased to 75%, which we feel is unacceptable in clinical practice. Very similar results were reported by Remer et al. [13]; however, Bae et al. [5] achieved an increase in sensitivity without a decrease in specificity. One possible explanation could be the smaller number of non-adenomas, mostly lung cancer metastases that seem to contain only limited numbers of negative voxels. A comparison of our result based on volume histogram analysis with the results of previous studies suggests that a volume histogram is not superior to single-image histogram analysis; a stronger influence is wielded by the portion of lipid-poor adenomas in the study sample, the portion of renal cell carcinoma metastases and lung cancer metastases among non-adenomas, and the degree of noise in a CT image.

Our work has several limitations. One of them is the retrospective nature of the study; in such a setting, the masses were examined over a long time period with a wide range of CT protocols dedicated to the assessment of chest and abdominal organs. In particular, it is necessary to discuss the slice thickness, which varied in different protocols. The slice thickness has a significant effect on the result of the histogram analysis, provided that the noise in the CT image increases in the thinner slice [18]. Therefore, it should be emphasized that noise is a more important parameter than the slice thickness itself, and we feel that the CT image noise was carefully assessed and quantified in our study. Therefore, we believe that although the protocols differed, the result of the histogram analysis was not significantly affected by noise. In our opinion, a prospective study of incidentally found masses is basically impossible, and our study thus reflects the daily clinical routine. It should be stressed that the PPV values in Table 4 should be understood only as values allowing a relative comparison among different combinations of slice thicknesses and thresholds within our study group. PPV is dependent on the prevalence of a certain pathology, and the prevalence of lipid-poor adenomas was artificially increased in our study group because of our inclusion and exclusion criteria; our PPV values, therefore, are also artificially increased and cannot be applied to a general population. It should also be admitted that in some of the patients in our study group, a correct diagnosis of adenoma, pheochromocytoma, metastasis, or carcinoma could be made on the basis of the clinical signs, laboratory blood tests, or routine CT by measuring the size of the lesion and native density or growth dynamics. Our article is radiologically focused and examines the technical possibilities of software CT image processing, tissue characterization, and fat component identification in adrenal tumors. The aim of our study was not to evaluate how many patients had a diagnostic uncertainty in their adrenal mass and to what extent CT histogram analysis would resolve this uncertainty. We are not sure to what extent the article would benefit from supplementing the data for part of the cohort, e.g., the results of hormonal tests in patients with pheochromocytoma, hyperaldosteronism, or Cushing’s syndrome. The question is how these results would contribute to the discussion of the possibilities of CT histogram analysis. Moreover, endocrinological data are not usually available in the other previous articles mentioned in this discussion. However, our own data regarding adrenal mass diameter have been published in another article, in which the study group partially overlaps with the study group in this manuscript [21].

## 5. Conclusions

Mean unenhanced attenuation represents a quick and simple tool for recognizing an adrenal adenoma that is suitable for clinical practice. A cut-off value of 10 HU provides the most specific measurement, but a cut-off value of 15 HU is also acceptable. The lower sensitivity of unenhanced attenuation could be increased by performing CT histogram analysis with a cut-off value of 10% of negative voxels.

## Figures and Tables

**Table 1:** Summary of mean unenhanced attenuation and percentage of negative voxels in adrenal masses.

	Adenomas					Pheochromocytomas					Carcinomas					Metastases					
	Med	Min	Max	Mean	SD **	Med	Min	Max	Mean	SD **	Med	Min	Max	Mean	SD **	Med	Min	Max	Mean	SD **	*p*
(a)																					
mean unenhanced attenuation (HU)	6	−23	50	9.6	14.3	34	17	80	33	8.5	30.5	24	41	31.6	5.2	32.5	9	56	32.4	8.8	<0.0001
(b)																					
percentage of negative voxels (%)	37.7	0	86.2	36.2	22.7	1.5	0	9.8	2.75	2.91	2.55	0	9.9	3.08	2.98	3.1	0	34.1	4.4	6.4	<0.0001
(c)																					
standard deviation of HU values *	16.9	7.1	29.5	17.5	5.1	15.9	5.9	24.6	15.6	3.9	14.6	8.3	21.7	15.1	3.9	16.6	6.6	27	16.5	4.1	0.0728

Med—median, Min—minimum, Max—maximum, SD—standard deviation, p—significance level calculated by Kruskal–Wallis test. * standard deviation of Hounsfield values measured inside each adrenal mass to quantify CT image noise, as demonstrated in Figure 1; ** standard deviation as a measure of the variability of the mean unenhanced attenuation; the percentage of negative voxels; and the standard deviation of HU values in the group of adenomas, pheochromocytomas; carcinomas, and metastases.

**Table 2:** Multiple pair comparisons between four histological groups.

	Unenhanced Attenuation	Percentage of Negative Voxels
adenomas vs. pheochromocytomas	<0.0001	<0.0001
adenomas vs. carcinomas	<0.0001	<0.0001
adenomas vs. metastases	<0.0001	<0.0001
pheochromocytomas vs. carcinomas	1.0	1.0
pheochromocytomas vs. metastases	1.0	0.876
carcinomas vs. metastases	1.0	1.0

**Table 3:** Summary of area under the curve results.

	AUC	Std. Error	Asymptotic 95% Confidence Interval	
			Lower Bound	Upper Bound
percentage of negative voxels	0.919	0.02	0.881	0.958
unenhanced attenuation	0.908	0.021	0.867	0.949

AUC—area under the curve, Std. error—standard error under the non-parametric assumption.

**Table 4:** Diagnostic test performance to distinguish adenomas from non-adenomas with different cut-off values.

	Cut-Off Values			
	Unenhanced Attenuation		Percentage of Negative Voxels	
	<10 HU	<15 HU	>10%	>5%
Sensitivity (%)	59.35	69.11	82.93	87.8
Specificity (%)	99.09	98.18	98.18	75.45
Positive predictive value (%)	98.65	97.7	98.08	80

HU—Hounsfield units.
